# Pennsylvania State Veterinary Medical Association

**Published:** 1900-02

**Authors:** J. Curtis Michener, Jacob Helmer

**Affiliations:** President; Secretary pro tem.


### SOCIETY PROCEEDINGS.

### PENNSYLVANIA STATE VETERINARY MEDICAL ASSOCIATION.

(Continued from page 59.)

Under the head of new business, Dr. Pearson presented a resolution on the death of Dr. John J. Smith, of Chambersburg, Pa., as follows:

*Whereas*, Our late fellow-member, Dr. John J. Smith, of Chambersburg, died on September 8, 1899, of anthrax contracted during the performance of his duty as a veterinarian; be it

*Resolved*, That this Association hereby expresses its sense of loss and grief upon the death of our late esteeemed colleague, and testifies to its appreciation of his heroic efforts to suppress the scourge that led to his death; be it further

*Resolved*, That these resolutions shall be spread on the minutes and that a copy shall be sent to the bereaved family.

Dr. Hoskins commented upon the character of Dr. Smith and showed the esteem in which he was held by the community in which he lived and labored as well as by his colleagues.

On motion by Dr. Rhoads it was unanimously decided to consider Dr. Smith a member of the Association when he died, although technically he was not, having allowed himself to be in arrears, on account of which his name had been placed among others on the list of members expelled.

On motion of Dr. Thomas B. Rayner, seconded by James B. Rayner, Dr. Pearson's resolution was adopted.

Dr. W. Horace Hoskins introduced a resolution as follows:

*Whereas*, The work of our State Livestock Sanitary Board has already attracted the favorable attention of many States and other countries, many of said States planning to follow the conservative and well resulting measures adopted by our State; and

*Whereas*, The successful results in dealing with the many serious problems covering the many infectious and contagious diseases occurring throughout our Commonwealth are largely to be attributed to the efficiency and wisdom of our fellow-member and State Veterinarian, Leonard Pearson; and

*Whereas*, His term of four years now about expiring finds us in our Commonwealth with well defined plans, with the united support of the people of our State, backed by an unbroken front of the profession in confidence of the safe direction of these all-important affairs; be it

*Resolved*, That we cordially indorse the work of our fellow-member, State Veterinarian Pearson, and recommend strongly his reappointment to our Governor, William A. Stone.

Moved by W. Horace Hoskins, seconded by Thomas B. Rayner. The resolution was adopted.

Dr. Rhoads brought before the Association the question of a relief fund for the wives and children of veterinarians who have lost their means of support. He asked for the views of the members upon the subject. Dr. Hoskins moved that the Chair appoint a committee of five to consider this important matter, in view of instituting a plan similar to the one in vogue in England. The motion was seconded by Dr. Rhoads. Dr. Pearson suggested that the committee hold the matter under consideration for at least six months, since it was an important question. Dr. Pearson held that an association such as the Relief and Protective Association of England would be useful in more than merely providing for the relief of indigent children, and cited the Hewson case in evidence, in which an important client was unjustly prosecuted by the S. P. C. A., but protected and cleared by the Relief and Protective Association. Dr. Hoskins's motion was carried.

Dr. Rhoads moved that the Chair between now and the next meeting appoint a committee to care for the records, paper, etc., that belong to the Association. An amendment to this motion was made by Dr. Hoskins, that the Corresponding Secretary put this subject in shape and present the same at the spring session, since the matter involved the creation of a new office. The motion with its amendment was carried.

The literary programme consisted of a series of interesting and instructive papers.

The first, entitled "A Veterinarian's Daily Observations," by Dr. Walker S. Phillips, was, in the doctor's absence, read by the Corresponding Secretary. The latter also read Dr. J. S. Butterfield's paper on "Œstris Ovis," and that of Dr. J. J. Repp on "Meat-inspection in Small Towns." Dr. D. C. Stanton, of Factoryville, talked on the subject of "Cattle," confining his remarks principally to contagious abortion and tuberculosis. Dr. Helmer read a paper entitled "Ethics of Consultation," and was followed by Dr. Pearson on "The Worth of the State Livestock Sanitary Board." Dr. John W. Adams spoke at length and forcibly upon the subject of "Meat-inspection."

Dr. Pearson offered a resolution on the death of Dr. Isaiah Michener, which was unanimously adopted. Dr. Hoskins offered a resolution as follows, which was unanimously adopted:

*Whereas*, The question of meat-inspection is now one of the daily problems confronting national, State, and municipal governments, and demanding a proper solution at the hands of those charged with the direction of public affairs in the care and protection of our people from the dangers that lie therein; and

*Whereas*, The creation of these positions is fraught with the danger that imperfectly educated and unfit persons seek and obtain positions of municipal meat-inspectors, thus robbing the people of the good that should flow therefrom; therefore, we earnestly commend the application of the civil service of our Federal Government in the selection of these officials; and

*Whereas*, The now conceded need of this protection in every city and borough of our State, and the fact that it would impose no hardships from a monetary point of view in increased taxation upon its people, as a tax of less than one cent per week to each inhabitant will provide for its maintenance in every city and borough, and in large cities can be made, through municipal abattoirs, a revenue-producing source as well as a saving factor to those engaged in the occupation of preparing meat foods for the people; be it

*Resolved*, That we earnestly commend this matter to the thoughtful consideration of city and borough officials and the public throughout our entire State.

Dr. Rhoads suggested that the Board of Censors, between now and the next meeting, consider the advisability of regarding members who have moved to other States as associate members, and that as such said members be exempt from the payment of the usual fees of regular members.

Dr. Hoskins thought the semi-annual sessions were not receiving the attention they should from the members. In the first place members failed to attend these sessions because there did not seem to be a sufficient object, but the doctor thought that the autumn meeting should be made a great object. It was the superior recruiting field. It brought the Association in contact with various local sections in the State, thus advertising and elevating the profession through the local press and through its influence directly upon members of the community. It afforded opportunity for profitable work for the attending members if advantage was taken of the opportunity. Dr. Hoskins was inclined to think that the autumn sessions were subordinated to the annual spring session in Philadelphia, which in that sense was an injury to the development of the fall session. Also, that too much time was given to recreation and entertainment, which was a serious hinderance to the success of the autumn session. The subject was discussed further by a number of the members present.

Dr. Rhoads called for a report from the Auditing Committee. Dr. Hoskins replied, asking the Association to receive the report as progressing. The amount due the Association is still a question. The completed report will be presented at the next meeting. He suggested providing the Treasurer with a book which should be a book of record.

On motion by Dr. Hoskins a vote of thanks was extended to Mrs. Helmer for her effort to entertain and provide the members each with a photograph which would always suggest the pleasant things of the autumn session in Scranton. Also, that a vote of thanks be extended to Mr. E. E. Loomis for the privilege of members to visit the interior of one of the coal mines. A vote of thanks was heartily extended to the local committees for their efforts to render the occasion a pleasant and profitable one.

Here a motion to adjourn prevailed, and the members departed.

In the evening a number of the gentlemen availed themselves of the opportunity to visit the mines.

The only drawback to a happy as well as a successful day was the rain which fell so abundantly.

J. Curtis Michener,
President.

Jacob Helmer,
Secretary pro tem.

